# Supplementary material for: A thalamic-hippocampal CA1 signal for contextual fear memory suppression, extinction, and discrimination
Source: Nat Commun. 2023 Oct 24;14:6758. doi: 10.1038/s41467-023-42429-6 (PMC10598272; doi:10.1038/s41467-023-42429-6)
Supplement: Supplementary file 1 — Supplementary Information [file 41467_2023_42429_MOESM1_ESM.pdf]

Supplementary Information

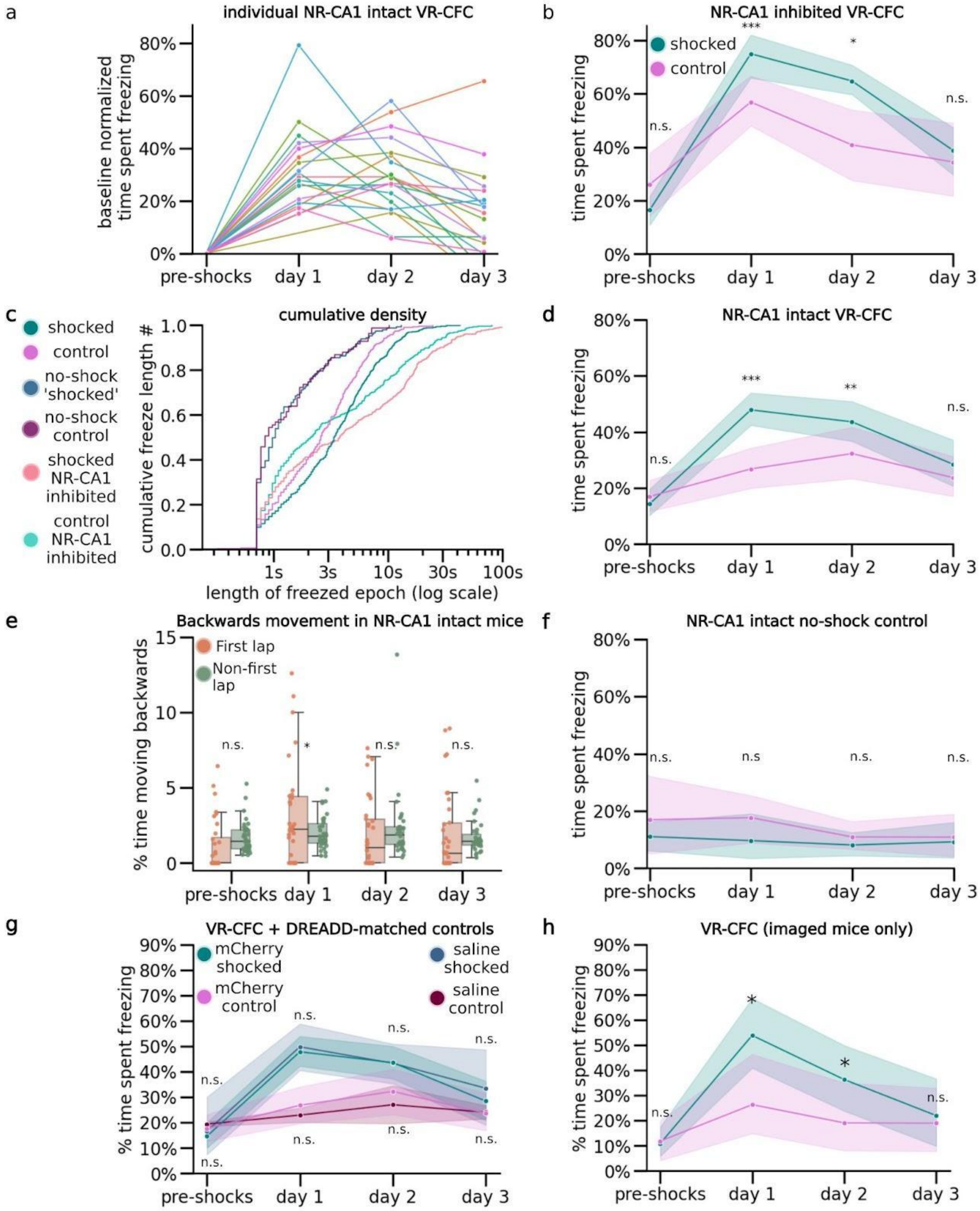

Supplementary Figure 1: Additional VR-CFC behaviors, controls and analyses

a, Percent time freezing normalized to baseline for each mouse in the NR-CA1 inhibited condition in the shocked context. **b,** Same as Fig. 2e, but plotted without normalization. Statistical comparison is between the shocked and control context on each day in the NR-CA1 inhibited condition (EMM P = pre-shocks: 1, day 1: 7.73e-5, day 2: 0.01, day 3: 1). **c,** Cumulative density plot of freeze lengths on retrieval day 1**. d,** Same as Fig. 2e, but plotted without normalization. Statistical comparison is between the shocked and control context on each day in the NR-CA1 intact condition (Wilcoxon Rank Sum, P = pre-shocks: 1, day 1: 8.49e-4, day 2: 4.71e-3, day 3: 1). **e,** Backward movement in NR-CA1 intact mice in the shocked context. Dots indicate average percent time spent moving backwards per freezing epoch, either in the first traversal (green), or other traversals (orange), boxplot indicates median, 25-75th interquartile range, whiskers include all data points. We observed instances of backwards movement behavior on the track. This was significantly more common on the first traversal of the track on retrieval day 1 than other traversals (Student's T, P = pre-shocks: 0.51, day 1: 2.30e-3, day 2: 0.30, day 3: 0.10). We interpret this back-movement as an attempt by the mouse to escape the context by 'backing' out, and classify it as a fearful behavior. **f,** Same dataset as Fig. 1f, but plotted without normalization. Statistical comparison is between the shocked and control context on each day (no shocks actually delivered), not compared to baseline (EMM P = 1 per day). **g,** DREADD construct does not alter behavior. Statistical comparison is between contexts for the NR-CA1 intact condition and the DREADD mCherry control condition (see Methods: DREADD experimental protocol. EMM P = 1). **h,** The freezing behavior of a subset of mice that were also imaged (N = 10). Statistical comparison is between the shocked and control context on each day, not compared to baseline (Wilcoxon Rank Sum, P = pre-shocks: 1, day 1: 0.01, day 2: 0.04, day 3: 1).

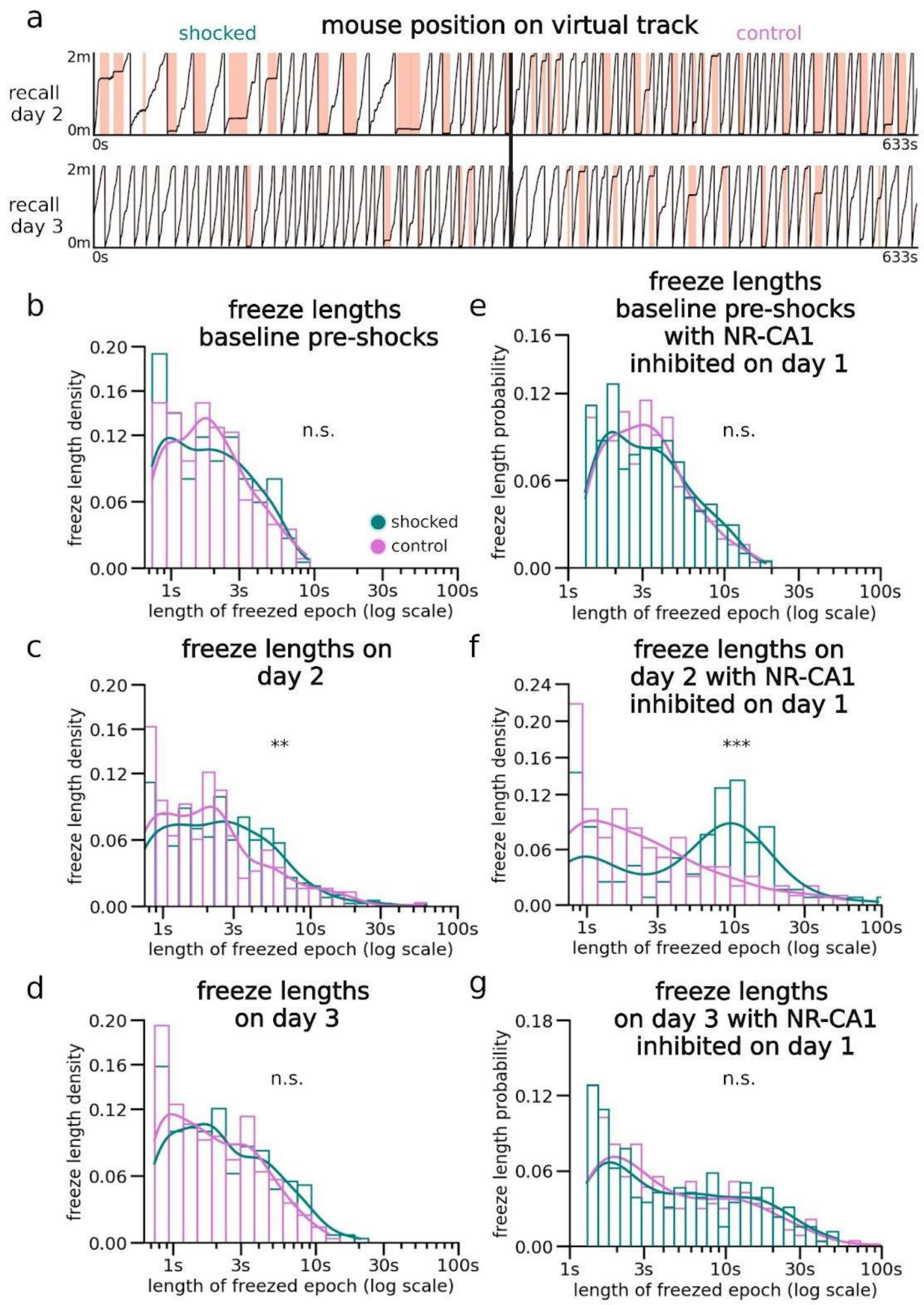
a
mouse position on virtual track
shocked
control
recall day 2
recall day 3
2m
0m
0s
633s
b
freeze lengths baseline pre-shocks
n.s.
shocked
control
freeze length density
length of freezed epoch (log scale)
c
freeze lengths on day 2
**
d
freeze lengths on day 3
n.s.
e
freeze lengths baseline pre-shocks with NR-CA1 inhibited on day 1
freeze length probability
f
freeze lengths on day 2 with NR-CA1 inhibited on day 1
***
g
freeze lengths on day 3 with NR-CA1 inhibited on day 1
1s
3s
10s
30s
100s

Supplementary Figure 2: Additional analyses of freeze lengths with and without NR-CA1 pathway inhibition

a, Example behavioral traces from retrieval day 2 (top) and retrieval day 3 (bottom) from the same example NR-CA1 intact mouse and displayed in the same fashion as Fig. 1d. **b-g,** Freeze lengths of individual freeze epochs calculated as in Fig. 1g, day and condition indicated on graph. All significance stars determined using Mann-Whitney U test (P = b: 0.47 c: 7.92e-3, d: 0.24, e: 0.31 f: 6.82e-3, g: 0.86).

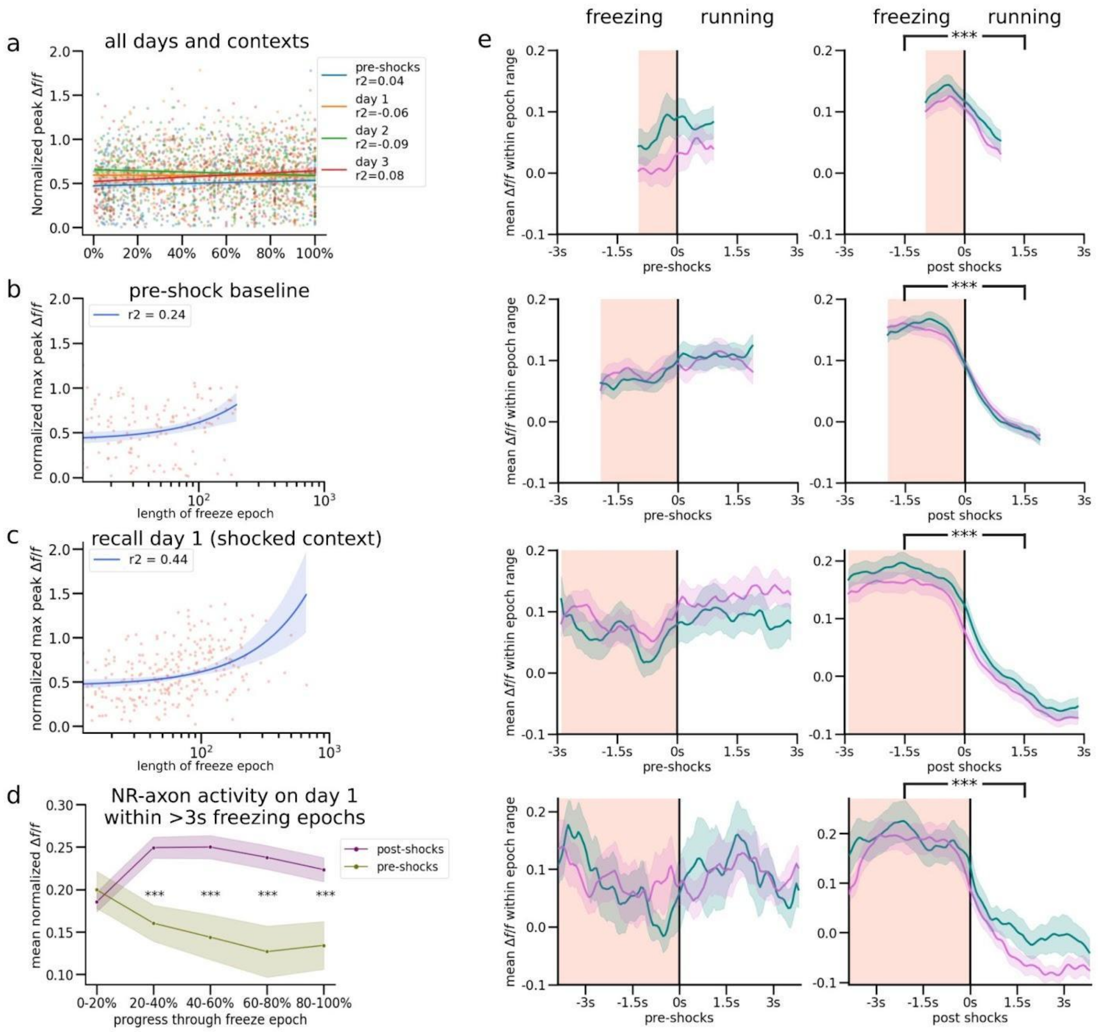

Supplementary Figure 3. Additional Comparisons of NR-CA1 axonal activity before and after CFC

a, The max activity of each axon per freeze epoch on each day in both contexts (dots, color-coded by experimental day), plotted against when it occurred within the freeze epoch (0% is the start of a freeze epoch, 100% is the end of a freeze epoch). A robust linear regression was then fitted to each day. This analysis indicates that the highest point of axonal activity within a freeze epoch can occur anywhere temporally within a freezing epoch. **b,** To test the impact of freeze length on max NR-CA1 axonal activity, we plotted the peak within each freeze epoch against the length of the freeze epoch in the NR-CA1 intact condition in the shocked context before shocks (behavior plotted in Fig. 1d) then fit the data with a robust quadratic regression (see Method Details: Statistics). The regression suggests a slight tendency for increased maximum amplitude in longer freezing epochs **c,** Analysis is the same as in 3b, but on retrieval day 1 in the shocked context. While the r^2 of the robust regression increased in the shocked context post-shocks compared to pre-shocks, since average freeze lengths also increased (as mice

freeze for longer epochs post-shocks), it is difficult to assert that fearful freezing is inducing changes in normalized max axonal peak as a function of time spent freezing from these analyses. **d,** Same analysis conducted as Fig. 2e, but on only a subset of longer pauses (3 s + in length) showing a similar average shape of activity throughout these freeze epochs to Fig. 2e. **e,** Analyses conducted the same as Fig. 2d, except the freeze and post-freeze running epoch window is restricted to different window lengths of 1-2 s, 2-3 s, 4-5 s, and 5-6 s, from top to bottom. All freezing epochs remained significantly elevated compared to running epochs post-shocks (Wilcoxon Rank Sum), with the same general underlying shape evident irrespective of the window chosen.

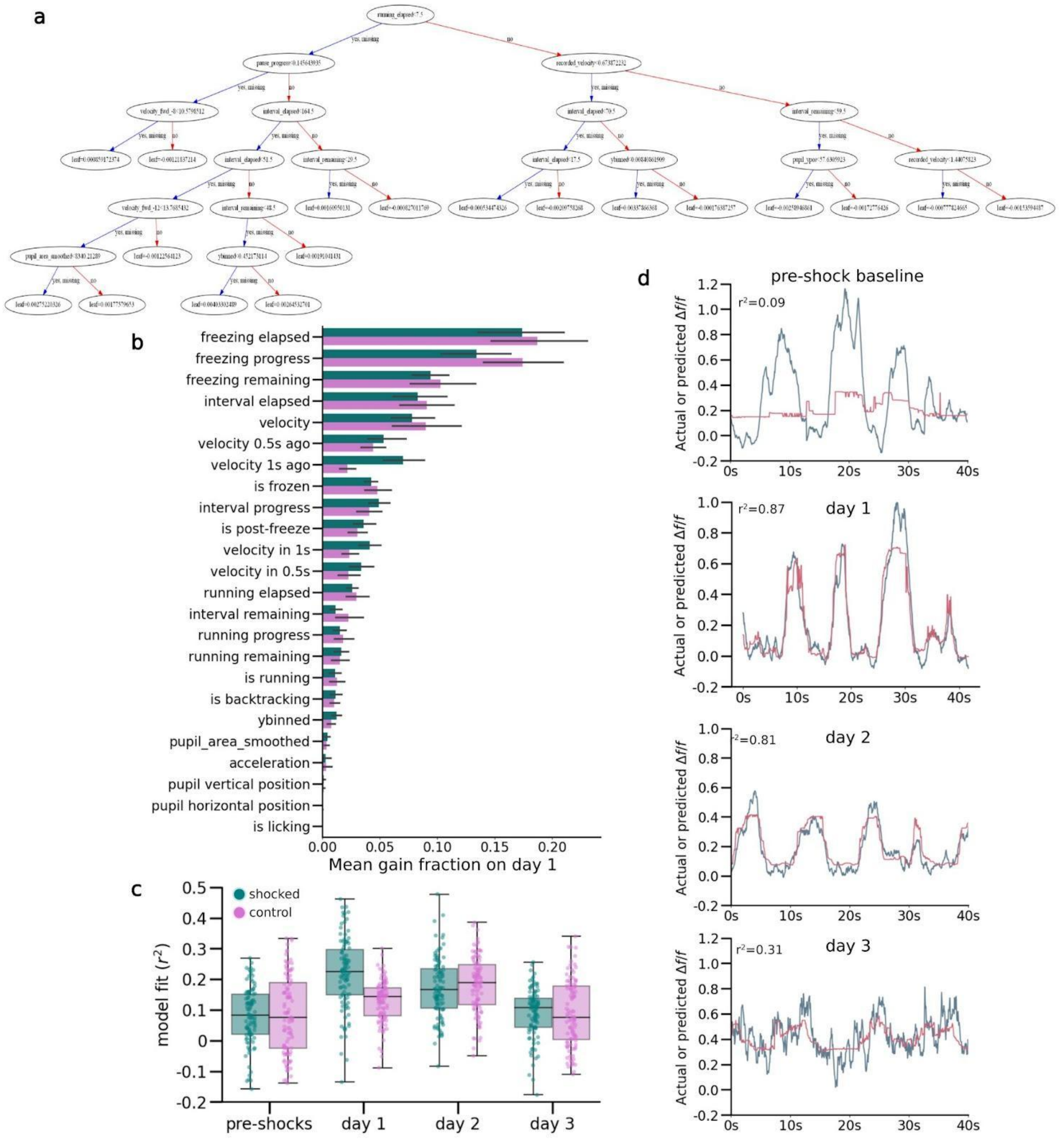

Supplementary Figure 4. Computational Model Details and Additional Examples

a, Real example of a model tree on retrieval day 1 in the shocked context from the same example mouse shown in Fig. 6d. **b,** Full ungrouped version of Fig. 6h. **c,** Same analyses as Fig 6g, except from a model that ran without differentiating and building models per individual mouse, but instead across all mice and differentiating only on day and context, demonstrating that high inter-mouse variability in axonal activity and amplitude led to decreased median r^2

goodness-of-fit model performance compared to inter-mouse modeling shown in Fig. 6g. **d,** Analyses same as Fig. 6c-f in the same mouse shown in Fig. 6c-f, but in the control instead of the shocked context.

Individual freezing epochs following CFC

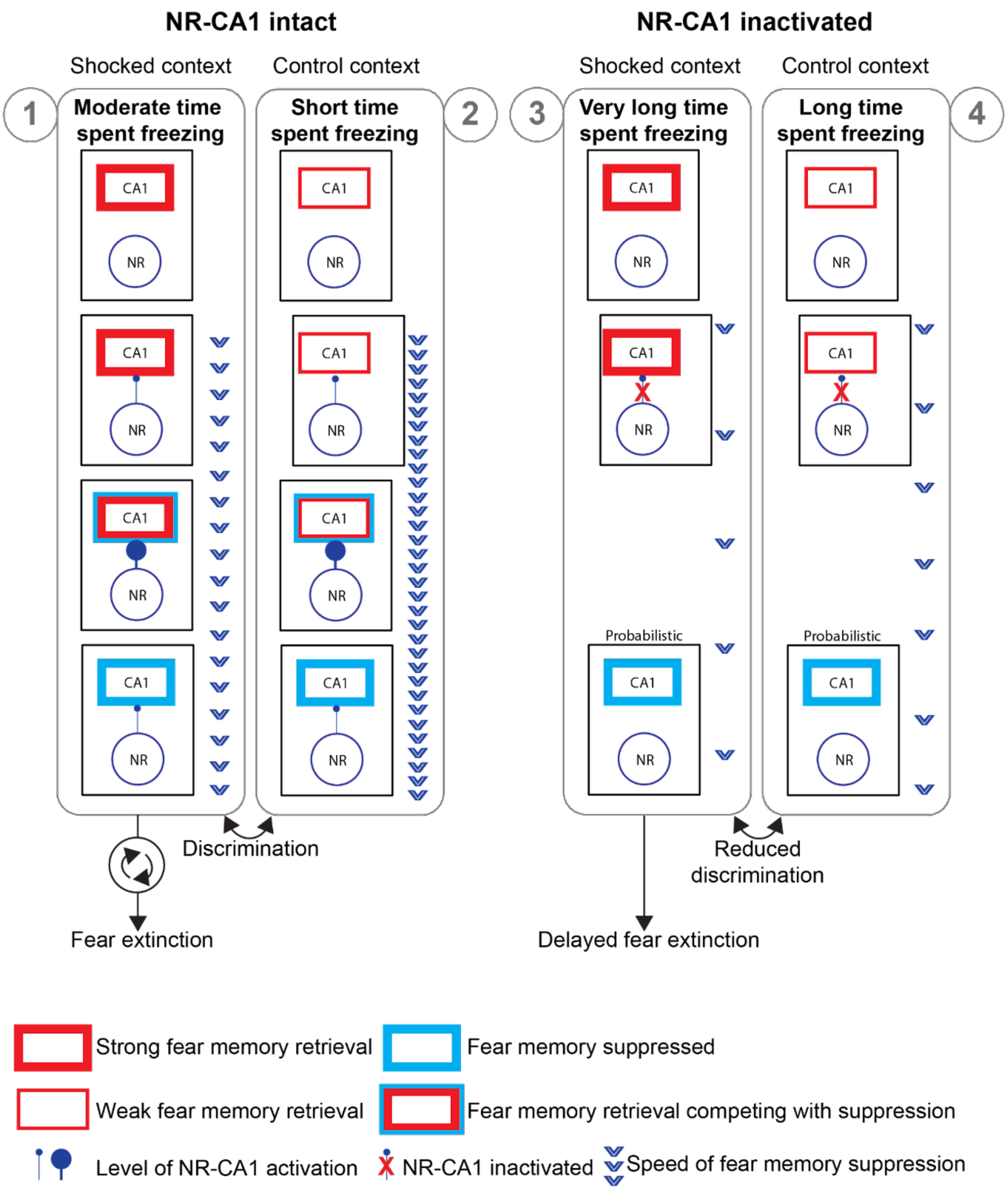

Supplementary Figure 5. Cartoon of Findings

The cartoon depicts contextual fear memory retrieval suppression in CA1 during freezing epochs post-CFC. From left to right the 4 conditions are a freezing epoch: (1) in the shocked context w/NR-CA1 intact; (2) in the control context w/NR-CA1 intact; (3) in the shocked context w/NR-CA1 inactivated; (4) in the control context w/NR-CA1 inactivated. In each condition, the strength of contextual fear memory retrieval is dependent on the context. Strong fear memory retrieval in CA1 is depicted by the thickness of the red box around CA1. The control context can also evoke freezing responses because the context has some similarities with the shocked context. Weak fear memory retrieval in CA1 is depicted by a thin red box. In the NR intact condition, the NR-CA1 pathway ramps up as soon as the animal is retrieving fear memories. After time, the NR-CA1 activity reaches maximum levels, providing a disrupting signal that promotes a state change in CA1, and the signal ramps down. This could indicate a decision-making phase during which fear memory retrieval has been suppressed, but the decision to move has yet to occur. It could be caused by a feedback signal from CA1 to NR, which anatomically exists but has an unknown role. This also occurs in the control context during freezing epochs (2), but it occurs faster because the CA1 is in a weak memory-retrieval state, making it easier for the NR-CA1 signal to act. The blue V's depict the speed of fear memory retrieval supressal in CA1. Repeated NR-CA1-induced suppression speeds up fear extinction. Further, the difference in speed at which NR-CA1 suppresses strong versus weak memory retrieval, i.e., in the shocked versus control context, allows for context discrimination. In the NR-CA1 inhibited condition, CA1 remains in fear memory retrieval state for longer because the NR-CA1 disruption signal is absent. It also reduces context discrimination, because even in the control context when animals are weakly-retrieving the fear memory, CA1 remains in this state. Lastly, because repeated NR-CA1-induced fear memory suppression does not take place during NR-CA1 inactivation, fear extinction is delayed.
